# Making inroads into improving treatment of bacterial vaginosis – striving for long-term cure

**DOI:** 10.1186/s12879-015-1027-4

**Published:** 2015-07-29

**Authors:** Catriona S. Bradshaw, Rebecca M. Brotman

**Affiliations:** 1 Melbourne Sexual Health Centre, 580 Swanston Street, Carlton, VIC 3053 Australia; 2 Central Clinical School, Monash University, Melbourne, VIC Australia; 3 Institute for Genome Sciences, University of Maryland School of Medicine, Baltimore, MD USA

**Keywords:** Bacterial vaginosis, Treatment approaches

## Abstract

Bacterial vaginosis (BV) is one of the great enigmas in women’s health, a common condition of unknown aetiology, which is associated with significant morbidity and unacceptably high recurrence rates. While it remains unclear whether BV recurrence is predominantly due to failure of current antibiotic regimens to eradicate BV-associated bacteria (BVAB) and biofilm, a failure of some women to re-establish a resilient *Lactobacillus*-dominant vaginal microbiota, reinfection from sexual partners, or a combination of these factors, it is inherently challenging to make significant inroads towards this goal. In this review, we will outline why BV is such a clinical and epidemiologic conundrum, and focus on several key approaches that we believe merit discussion and clinical research, including strategies to: i) prevent reinfection (partner treatment trials), ii) boost favourable vaginal *Lactobacillus* species and promote a *Lactobacillus*-dominant vaginal microbiome (hormonal contraceptive and probiotic trials) and iii) disrupt vaginal BV-associated biofilm.

## Introduction

Bacterial vaginosis (BV) is one of the great enigmas in women’s health, a common condition of unknown aetiology, which is associated with significant morbidity and unacceptably high recurrence rates. In this review, we will outline why BV is such a clinical and epidemiologic conundrum, and propose key research priorities that focus on the common goal, the elusive sustained cure.

### Background

BV is the most common vaginal infection in women of reproductive age. Prevalence estimates range from 12 % in Australian women [1], to 29 % in North-American women [2, 3], and greater than 50 % in women in East/Southern Africa [4]. BV has been associated with serious and costly reproductive and obstetric sequelae, increasing women’s risk of pre-term delivery, low birth weight, miscarriage and pelvic inflammatory disease [5]. These sequelae have considerable implications for healthcare expenditure, with the population attributable risk of BV for pre-term delivery in the US estimated over a decade ago to be 30 %, at a cost of USD 1 billion per annum [5].

Longitudinal studies have demonstrated that BV is associated with an approximate 2-fold increased risk of acquiring sexually transmitted infections including chlamydia, gonorrhoea, herpes simplex type 2 and HIV infection [6–11], and increases the risk of co-infected women transmitting HIV to their male partners [12]. Although significant proportions of women with BV appear to be asymptomatic, over 50 % experience an unpleasant vaginal malodour and discharge, with qualitative studies showing BV is associated with moderate-severe impact on self-esteem, sexual relationships and quality of life in women who have sex with men (WSM) and women who have sex with women (WSW) [13].

While the aetiology and pathogenesis of BV is not completely understood, BV is characterised by depletion of key *Lactobacillus* spp., high bacterial species diversity and increased loads of facultative anaerobes such as *Gardnerella vaginalis*, *Atopobium vaginae* and other fastidious BV-associated bacteria (BVAB) including *Megasphaera*, *Sneathia* and *Clostridiales* spp. compared to healthy controls [14–16]. Most vaginal *Lactobacillus* spp. provide broad-spectrum protection against pathogens through production of potent antimicrobial molecules, bacteriocins and lactic acid [17–19], with the latter acting as a broad-spectrum bactericide and virucide, and maintaining vaginal pH between 3.5 and 4 [20, 21]. Lactic acid appears to possess antimicrobial activity beyond acidity alone, by disrupting the integrity of bacterial cell membranes and stimulating innate immunity in the presence of bacterial lipopolysaccharide [20–22]. Recent work has also demonstrated that lactobacilli can interfere with the ability of pathogens such *Trichomonas vaginalis* to adhere to host cells [23]. Overall there is a significant body of evidence indicating most *Lactobacillus*-dominant vaginal microbiomes, with the exception of *L. iners* [24–29], are optimal for maintaining vaginal and reproductive health.

Recent work has identified a vaginal wall biofilm in women with BV, dominated by *G.vaginalis* and *A.vaginae*, that is absent in healthy controls [30, 31]. While it is thought this biofilm may contribute to the low success rate of current antibiotic therapies, the role of the biofilm in BV pathogenesis is not clear as *in vivo* vaginal epithelial cells are shed rapidly throughout the menstrual cycle [32]. BV research is further impeded by the fact that primate and other animal models are not optimal for the study of the cervico-vaginal microbiota because the vaginal pH is higher (>5) and the resident microbiota differ from that of humans [33–37]. There are, for example, high proportions of streptococci in mice [35] and *Corynebacterium* in the guinea pig [34]. Humans also have significantly lower bacterial richness and diversity estimates compared to non-human primates, and the relative abundances of lactobacilli are significantly lower in non-human primates [33].

While short term BV cure rates following first line recommended therapies (5–7 days of metronidazole or clindamycin) approach 80 % [38], failure rates in excess of 50 % occur within 6–12 months [39, 40]. Higher baseline loads of several BVAB have been associated with increased risk of recurrence [41], and some data suggest BV-associated biofilm rapidly re-accumulates following antibiotics [31]. However, strategies such as suppressive antibiotic regimens that attempt to eradicate persistence of BVAB, have not achieved sustained high long term cure [42, 43].

**Fig. 1:**
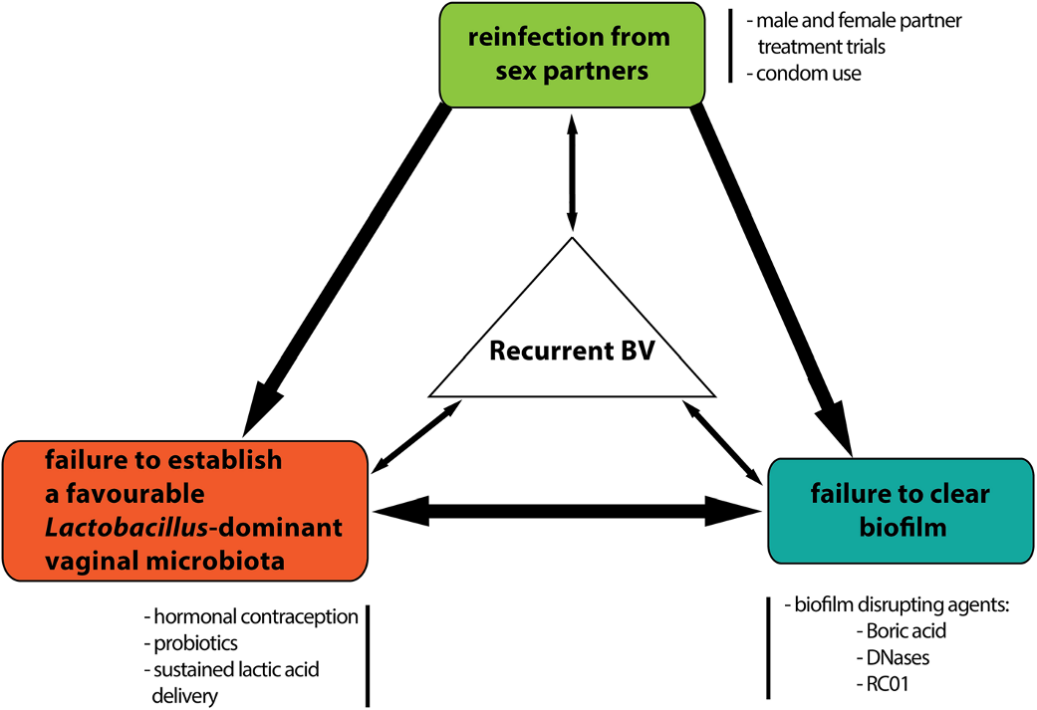
BV recurrence and potential therapeutic strategies

While it remains unclear whether BV recurrence is predominantly due to failure of current antibiotic regimens to eradicate BVAB and biofilm, a failure of some women to re-establish a resilient *Lactobacillus*-dominant vaginal microbiota, reinfection from sexual partners, or a combination of these factors, it is inherently challenging to make significant inroads towards this goal, Fig. 1. We will focus this review on several key approaches that we believe merit discussion and clinical research, including strategies to: i) prevent reinfection (partner treatment trials), ii) boost favourable vaginal *Lactobacillus* species and promote a *Lactobacillus*-dominant vaginal microbiome (hormonal contraceptive and probiotic trials) and iii) disrupt vaginal BV-associated biofilm.

### Strategies to address potential reinfection (partner treatment trials)

BV has been strongly associated with sexual activity in epidemiological studies. Women with BV have an earlier median age of sexual debut than women without BV [44], and BV has been associated with increased numbers of recent and lifetime sexual partners and inconsistent condom use by meta-analysis [45]. A study of young female university students found BV to be absent in women with no history of sexual activity, to be uncommon in women who had engaged only in non-coital sexual activities and to be significantly associated with penile-vaginal sex [46]. Prior studies identifying BV in “virgins” had limited questioning to history of penile-vaginal sex [47, 48]. The concept that BV may be sexually transmitted has been controversial for many years due to difficulties encountered in determining its aetiology, absence of corresponding disease in males, and failure of male partner treatment trials to reduce recurrence [49, 50]. A recent systematic review by Mehta concluded that insufficient power, use of non-standard treatment regimens, no measures of adherence, and poor retention, rendered the findings of past partner treatment trials inconclusive by current standards, and recommended larger trials using recommended therapies be conducted [50]. Further support for sexual transmission of BV includes published data which indicates that the poor long-term performance of recommended therapies may at least partly be attributed to post-treatment sexual behaviours. Women exposed to an ongoing partner pre- and post-treatment were at a 2–3 fold increased risk of BV recurrence after adjusting for sexual frequency, condom use and hormonal contraception in two studies [40, 51], and several, but not all studies, found inconsistent condom use and unprotected penile-vaginal sex to be associated with recurrence [51–53]. Male carriage of *G.vaginalis*, an organism implicated in the development of BV [54], is commonly reported [55–57]. Pyrosequencing of the microbiota of the coronal sulcus and distal-urethra in young males has also showed these sites to be colonised by BVAB, and the composition of the sulcus microbiota to be influenced by circumcision and sexual activity [58]. Male circumcision was not only associated prospectively with a significant reduction in penile anaerobes, including BV-associated genera [59], but a secondary analysis within a male circumcision trial showed wives of circumcised males had a reduced risk of BV (adjPRR = 0.60; 95 % CI = 0.38-0.94) compared to wives of controls [60]. While there are few studies examining BV-associated biofilm in men, biofilm has been detected in male urine and semen, and more commonly found in the male partners of females with BV than healthy controls [61]. Overall this body of evidence provides increasing support for the hypothesis that BV is sexually transmitted between men and women.

International BV research has had a strong focus on women who predominantly have sex with men. However, WSW also experience BV and are in need of relevant clinical and epidemiological research. A number of studies have confirmed BV to be prevalent in WSW with estimates ranging from 25 to 52 % [62–66]. Female same-sex partnerships provide an important model to understand the pathogenesis and transmissibility of BV, as unlike in male–female partnerships, BV can be diagnosed in both partners. BV has been strongly associated with sexual risk factors in WSW including: increased number of female partners, a female partner with BV and receptive oral sex [63, 65–68]. Female couples in monogamous relationships have high concordance of Nugent category [62, 63, 65, 66, 68], and share *Lactobacillus* strain types [69]. In a recent community-based cohort study of 298 WSW, incident BV was significantly associated with exposure to a new female sexual partner and a female partner with BV symptoms [68]. In contrast, co-enrolled couples who were BV-negative at enrolment had a very low risk of incident BV over two years, and their vaginal microbiota remained aligned, stable and within the “normal” Nugent category. This study provides compelling evidence for dynamic exchange of vaginal bacterial species between female partners, and extends our understanding of the influence of sexual behaviours and relationships on the vaginal microbiota and risk of BV.

Collectively, published clinical and epidemiological data suggests sexual transmission of BV is at least likely to be contributing to the development of BV and to post-treatment recurrence. These data provide broad support for the need to repeat and fund sufficiently powered male and female partner treatment trials to determine if this strategy reduces BV recurrence and associated sequelae. There is currently one registered male partner treatment trial enrolling couples in North America in which men are randomized to oral metronidazole versus oral placebo and these data are eagerly awaited (ClinicalTrials.gov Identifier: NCT02209519). Trials involving the use of topical in addition to oral agents are planned, and may be necessary to eradicate cutaneous carriage of BVAB from the penile skin. Female partner treatment trials, while clearly needed, are logistically more challenging as randomization of symptomatic female partners to treatment or placebo may not be acceptable or ethical; the design of such studies will clearly need careful thought and innovation.

### Strategies to boost favourable *Lactobacillus* spp.: vaginal probiotics and hormonal contraceptives

**Fig. 2:**
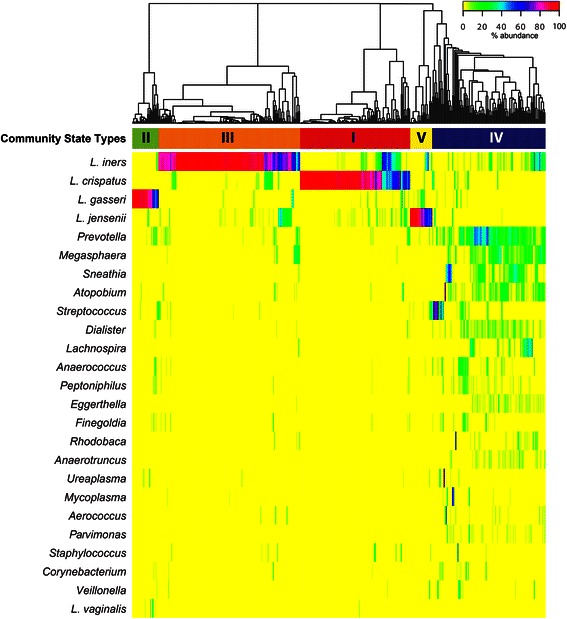
Heatmap showing the distribution of bacterial taxa found in the vaginal microbial communities of 394 reproductive-age women in the U.S. Adapted with permission from *Proceedings of the National Academy of Sciences of the United States of America* [70]

**Fig. 3:**
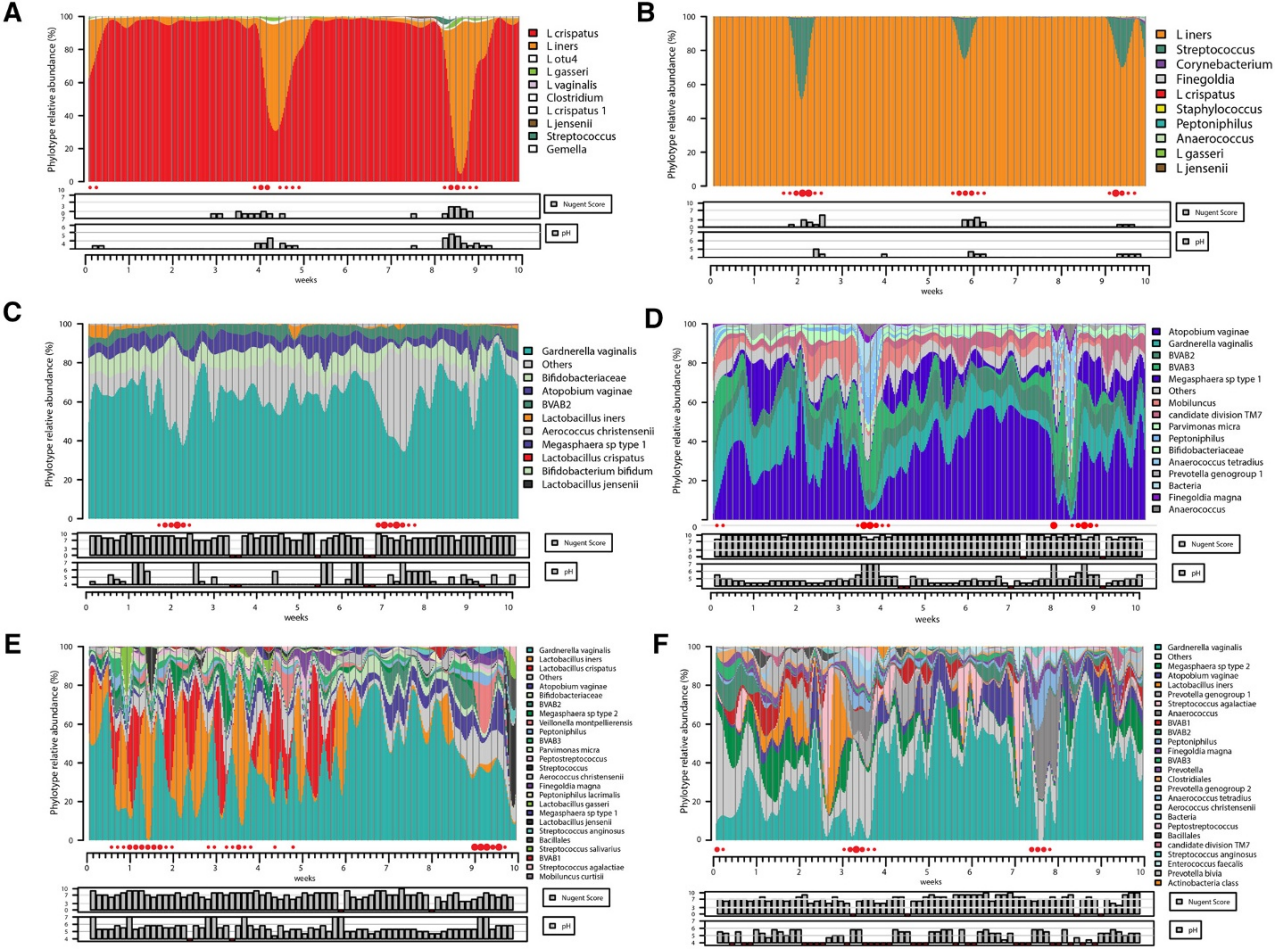
Daily temporal dynamics of vaginal bacterial communities in six women over a 10-week period. The relative abundance of each bacterial taxa is depicted as interpolated bar graphs. The bacterial taxa are indicated on the right of each bar graph with color codes. Daily Nugent Gram stain scores (range 0–10) and pH (range 4–7) are indicated below the graph. Red solid circles represent menstruation. Missing pH values are indicated by red box, otherwise pH is in line with a value of 4. Missing Nugent scores are also indicated by the red box, otherwise the score is in line with 0. The figure illustrates that the top four participants (**a**, **b**, **c**, **d**) carry highly stable communities dominated by *L. crispatus* (**a**), *L. iners* (**b**) and non-*Lactobacillus* dominated communities (**c** and **d**). Women **e** and **f** experienced unstable communities with both high Nugent scores and pH. Adapted with permission from *Microbiome* [78]

Surveys of the vaginal microbiota have found there are fundamental differences in the microbial diversity of vaginal communities present in reproductive age women [70–76]. In one U.S. study of 394 women, Ravel *et al.* characterized the vaginal microbiota using 16S rRNA gene analysis amplified from whole genomic DNA isolated from vaginal swabs [70], Fig. 2. Five vaginal microbiota groupings, termed community state types (CSTs), were identified. Four CSTs were dominated by one of four *Lactobacillus* species, while the fifth was depleted of *Lactobacillus*. The latter CST contained high proportions of anaerobic bacteria, resembling BV. The frequencies of each CST varied by ethnicity, and African American and Hispanic women were more likely to be *Lactobacillus*-depleted. Longitudinal studies have also demonstrated that some women experience frequent and rapid fluctuations in the composition of the vaginal microbiota, while in others, the microbiota are remarkably stable [77–79], Fig. 3. These surveys are particularly relevant to the development of probiotics, which aim to reseed dysbiotic microbiomes.

Probiotics that have been evaluated in studies have contained a range of *Lactobacillus* spp. (*L. acidophilus, L. gasseri. L. fermentum. L. rhamnosus* and *L. crispatus),* not all of which are endogenous to the vagina. In 2009, a Cochrane review found no conclusive evidence to recommend for or against probiotic use for the treatment of BV [80]*.* However, a recent body of research has begun to rigorously evaluate the efficacy of intravaginal and oral probiotic formulations to treat BV and to restore a *Lactobacillus*-dominant microbiota [80–83]. In a study of 90 women without BV who used the intravaginal delivery of *L. crispatus* CTV-05, Antonio et al. reported good CTV-05 colonization rates (59 %) at 28 days [83]. Subsequently, Ngugi et al. reported on the use of CTV-05 in 24 women with BV [84] and reported 44 % of BV cases were colonized with CTV-05 at 28 days, and cases not colonized had higher median concentrations of BV-associated bacteria. Those who were colonized had significant reductions in *Atopobium vaginae*, a bacterium associated with BV. Importantly, vaginal intercourse was found to significantly impair CTV-05 colonization in both studies, and endogenous *L. crispatus* also reduced the CTV-05 colonization rate [83, 84]. Clinical trials to assess the efficacy of CTV-05 for BV are in progress. A study of another intravaginal probiotic (containing *L. bre*vis, *L. salivarius* subsp. salicinius, and *L. plantarum*) found 61 % did not have BV by Amsel and Nugents criteria at 2 weeks compared to 19 % in the placebo group [85]. Using two well-characterized strains (*Lactobacillus rhamnosus* GR-1 and *Lactobacillus fermentum* RC-14), delivered by oral capsules, Reid *et al.* [86] reported a significant increase in vaginal lactobacilli on Gram stain and culture at day 28 and 60 in a randomized, placebo-controlled trial of 64 healthy women. Martinez *et al.* combined antibiotics and probiotics in a study of 32 women randomized to oral capsules of *Lactobacillus rhamnosus* GR-1 and *Lactobacillus reuteri* RC-14 following 2 g of tinidazole, and reported a BV cure rate at four weeks of 87 % in the *Lactobacillus* group compared to 50 % in the tinidazole-only group [87].

Although recent results are encouraging, these are relatively small studies with varying exclusion criteria and short follow-up. The overall conclusions of the recent Cochrane review and the latest U.S. CDC treatment guidelines are that current evidence does not yet support the addition of any available *Lactobacillus* formulations or probiotics as an adjunctive or replacement therapy in women with BV [80, 88]. There clearly remains a need for larger, well-designed, double-blind, placebo-controlled trials of various probiotic formulations, in combination with, or in comparison to, standard treatments. Probiotic trials need to standardize their methods for evaluating BV and given the high recurrence rates reported in trials that extend follow up to 6–12 months [40, 42], studies with more prolonged of observation post-treatment are important. As some evidence suggests a woman’s endogenous microbiota may impact on probiotic success rates, it is likely that future therapeutic approaches will need to include personalized probiotic or prebiotic recommendations based on a woman’s individualized temporal CST pattern.

**Fig. 4:**
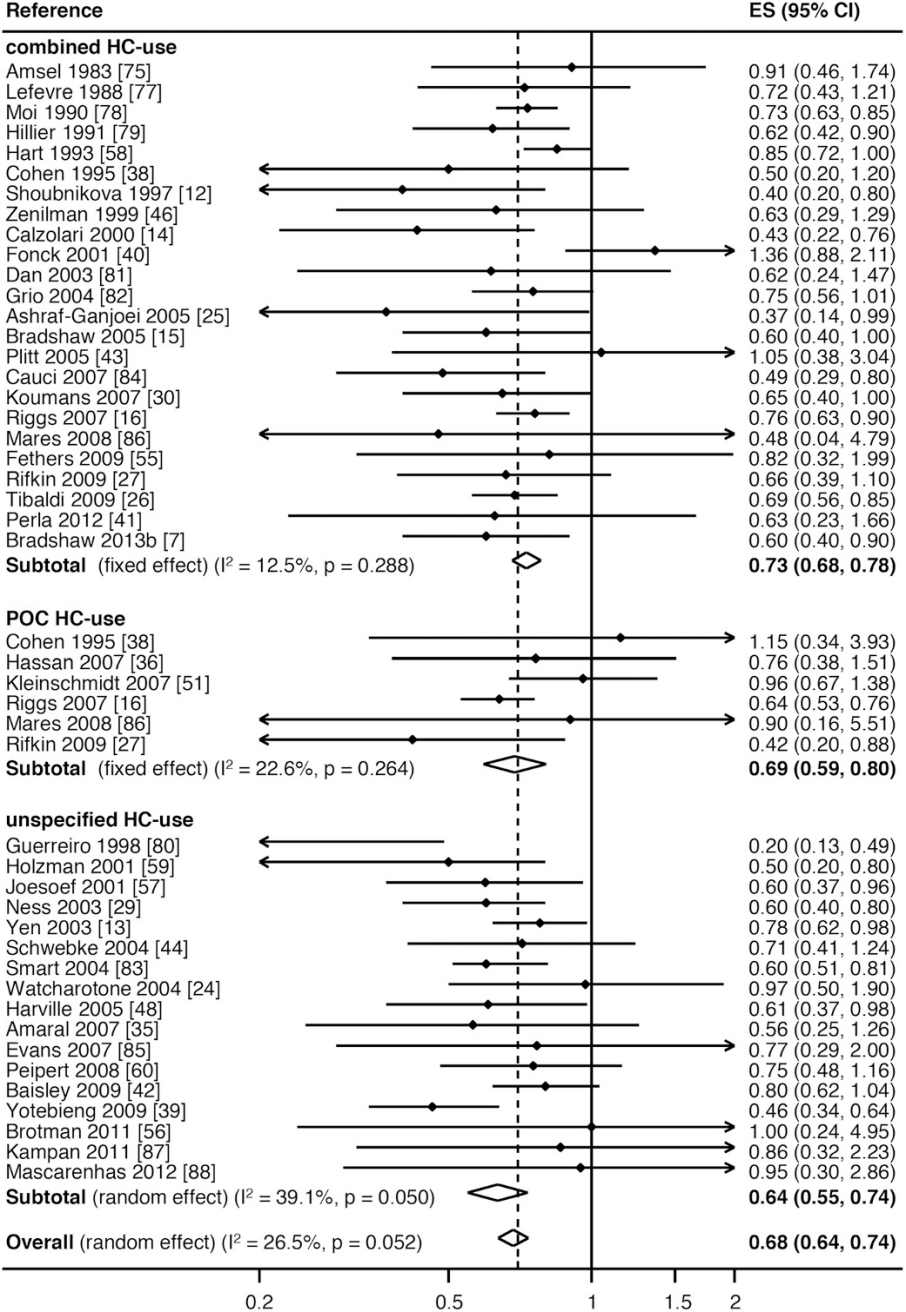
Meta-analysis of hormonal contraception and prevalent BV. Figure first published in *PLoS One* [93]

**Fig. 5:**
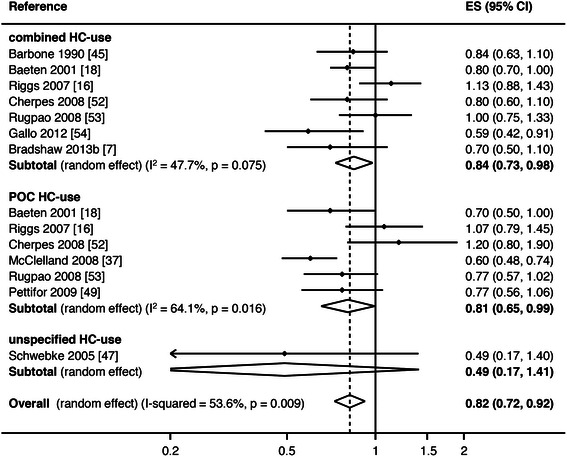
Meta-analysis of hormonal contraception and incident BV. Figure first published in *PLoS One* [93]

Another adjunctive approach that is being investigated to try and reduce BV following antibiotic therapy is hormonal contraception (HC). Epidemiological data, including a meta- analysis, suggest that oral contraceptive pills (OCP) are associated with a decreased risk of prevalent and incident BV, Figs. 4 and 5 [51, 89–93]. This meta-analysis also suggested that progestin-only oral contraceptives (POC) reduce BV to a similar magnitude seen with combined hormonal contraceptives [93]. Data on the influence of contraceptive vaginal rings on the local microenvironment are still sparse [94, 95], and there is inconsistent evidence on how copper and hormonal intrauterine devices (IUD) affect the vaginal microbiome [90, 96–98]. Overall, where data on HC are conflicting, it is most likely due to a number of factors including the heterogeneity of approaches used to diagnose BV, the formulation, regimen compliance and duration of HC use, and the observational nature of the studies. Sexual behaviours, and particularly condom usage, may also confound the relationship between contraceptive method and BV [97].

The effect of HC on BV and the vaginal microbiota is likely mediated by sex hormones and their effects on the genital microenvironment and immune response. Adequate estrogen levels increase glycogen production in vaginal epithelial cells, and glycogen is broken down by human alpha-amylase into metabolites used by *Lactobacillus* spp. to thrive and produce lactic acid [99]. HC, especially those containing progestins, also inhibit uterine bleeding and reduce menstrual loss. Menstruation has been strongly correlated with abnormal vaginal microbiota [77, 79, 100], perhaps due to blood being a more favourable environment for growth of iron-requiring BV-associated bacteria. HC may also promote a favourable anti-inflammatory cytokine milieu that facilitates BV clearance, either independently or mediated by the microbiota.

Despite >50 years of widespread use, the effect of HC on mucosal responses and the vaginal microbiota are still incompletely understood. Few studies have fully assessed changes in the vaginal microbiome after initiation or cessation of HC. For a more thorough examination of the complex relationship between HC and the vaginal microenvironment, we refer the readers to a review by Achilles *et al.* [101] and meta-analysis by Vodstricil *et al.* [93]. Future work will clarify whether use of specific HCs are definitively associated with a reduced risk of BV. Investigators are also evaluating lactic acid in the form of a vaginal gel and vaginal ring to determine if it prevents BV, which may be a future therapeutic option alone or in combination with HCs.

### New strategies to disrupt BV-associated vaginal biofilm

As increasing evidence has emerged that BV may be a biofilm-associated disease, an interest has emerged in agents that are primarily aimed at disrupting BV-associated biofilm [102]. It may be necessary to breakdown biofilm to achieve optimal efficacy of antimicrobial or probiotic therapies. Potential candidates that have been investigated include: vaginal boric acid, DNases, retrocyclins, octenidine and some naturally occurring antimicrobials (subtilosin, ploy-L-lysine, lauramide arginine ethyl ester) [103]. Boric acid and octenidine are the only compounds that have been used in human studies, while the remainder have been evaluated in *in vitro* studies against a *G.vaginalis*-biofilm [104, 105].

Boric acid (H_3_BO_3_), a white, odorless powder, was first used by Lister as a topical antiseptic in 1873 [106], and has been used in the treatment of vulvovaginal candidiasis [107–112] and *Trichomonas vaginalis* [113]. Vaginal boric acid has bacteriostatic and fungistatic action, yet its mechanism of action is unknown [114]. Boric acid may work on BV through decimation of the vaginal biofilm or change in the acidity of the vagina, while the fungistatic activity may be attributed to fungal cell wall penetration or disruption of the fungal cell membrane [115]. Intravaginal boric acid has a long history of clinical use in the treatment of vaginitis and is regarded as a safe secondary regimen [104, 111]. Blood boron analyses indicates little absorption from the vagina [116], and boric acid does not appear to cause adverse changes on cervical cytology [109]. Vulvovaginal burning, watery discharge, erythema, as well as male dyspareunia, are the most frequently reported adverse events reported by a minority of women [109, 115, 117]. Reichman et al. reported on a study of 7 days of oral nitroimidazole administered to 58 women with recurrent BV. Imidazole therapy was followed by 21 days of intravaginal boric acid (600 mg/day) and 16 weeks of suppressive metronidazole gel maintenance therapy if in remission at 21 days [104]. Patients reported symptom improvement and a high cure rate (87 %) at 2–3 months on treatment, however, by 38 weeks off treatment the BV recurrence rate was 50 %. Current ongoing research is examining how boric acid-based treatments, enhanced with an EDTA excipient to boost antimicrobial activity, may be active against the vaginal biofilm and be a potential candidate for BV treatment [118].

Swidsinski recently reported on the use of the topical antiseptic, octenidine dihydrochloride, for BV-associated biofilm [119]. Octenidine has broad spectrum antimicrobial activity and has been found to be highly effective against biofilms in oral, wound and orthopaedic implant infections [120–123]. Twenty-four patients with recurrent BV were treated with a 7 day intravaginal course of octenidine dihydrochloride spray, and if they failed treatment or recurred within 6 months, they were retreated with a 28 day regimen followed by weekly applications for 2 months [119]. Biofilm was evaluated by fluorescence *in situ* hybridization on voided vaginal epithelial cells. While early cure rates looked promising after 7 days of topical octenidine (87.5 %), six month recurrence rates were high (66.6 %). Repeated treatment for 28 days led to an overall cure rate of 75 %, however, complete bacterial resistance to octenidine occurred in a subset of women. Overall, while initial cure rates looked promising, the efficacy of prolonged and repeated treatment was poor and bacterial resistance emerged in a significant proportion of women.

Another novel strategy involves the use of DNAse which targets extracellular DNA (eDNA) [105]. *G. vaginalis* biofilms contain eDNA which is integral to their structural integrity. Enzymatic disruption of eDNA specifically inhibits biofilm formation and established biofilms. *In vitro* studies show that low concentrations of DNase and metronidazole have improved efficacy against *G. vaginalis* biofilm compared with either agent alone, presumably because DNase frees *G. vaginalis* from the biofilm and renders bacteria more susceptible to the antibiotic. Other *in vitro* studies have included evaluation of an anti-HIV microbicide candidate RC-101, a synthetic retrocyclin, which is an antimicrobial peptide with antiviral activity. RC-101 has been shown to potently inhibit the activity of vaginolysin, a protein toxin produced by *G. vaginalis*, and the formation of *G. vaginalis* biofilms *in vitro,* without affecting *Lactobacillus* spp. [124, 125]*.* Vaginolysin inhibition has been proposed as a potential strategy for BV treatment and prevention [124, 125]. While RC 101 inhibited the formation of GV biofilms, it is not clear if the mechanism is predominantly mediated through vaginolysin inhibition, or another substance involved in biofilm assembly [124, 125]. RC101 may be a candidate for human BV studies. Lastly, a novel area of research involves investigation of agents that inhibit quorum sensing. Quorum sensing is used by some bacterial species such as *Pseudomonas aeruginosa* and *Staphylococcus epidermidis* to co-ordinate expression of genes involved in virulence, biofilm formation and pathogenicity [126]. Quorum sensing inhibitors have not yet been evaluated in human studies or BV, but have been shown to be active *in vitro* against biofilms produced by *Pseudomonas aeruginosa* and *Staphylococcus spp*. [126, 127]. Clearly the identification of novel therapeutics that could be used safely in human trials alone or as adjunctive therapies to antibiotics for biofilm-inhibition and disruption is an emerging area of research. For a thorough review of the potential contribution of biofilm to treatment failure and recurrence in BV, we refer readers to an excellent recent publication by Muzny and Schwebke [102].

## Conclusion

No major therapeutic advances have been made in over 20 years that have achieved significant improvements in BV cure rates. We have only begun to understand the importance of the vaginal microbiome and how changes in its composition and function can affect women’s health. Approaches that aim to restore a woman’s “healthy” vaginal microbiome and maintain homeostasis are much needed to prevent recurrent BV and its sequelae, including transmission and acquisition of HIV and adverse obstetric outcomes, such as preterm birth. While it is fundamentally unknown if BV recurrence is principally due to persistence of BVAB and biofilm, a failure to re-establish a favourable *Lactobacillus*-dominant vaginal microbiota, reinfection or a combination of these factors, it is challenging to make progress. While evaluating many current and proposed therapies it is important to consider the possibility that reinfection from partners may also be impacting on our ability to determine the efficacy of these agents. Clinicians and researchers in the field consider it an urgent priority to develop new and innovative approaches to the management of BV in order to achieve high and sustained long-term cure rates, to develop effective prevention strategies and to reduce BV-associated sequelae. In order to achieve sustained cure it is possible, even likely, that we may need an approach that combines a number of these strategies such as use of antibiotics with biofilm-disrupting agents and partner treatment.
